# Supplementary material for: Physiotherapy and Exercise Management of People Undergoing Surgery for Lung Cancer: A Survey of Current Practice across Australia and New Zealand
Source: J Clin Med. 2023 Mar 9;12(6):2146. doi: 10.3390/jcm12062146 (PMC10051547; doi:10.3390/jcm12062146)
Supplement: Supplementary file 1 [file jcm-12-02146-s001.zip › Supplementary Material 1.pdf]

Supplementary File 1: Survey Instrument

The following is an example survey instrument exported directly from the online platform. In order to demonstrate an example of the original survey, this example activates all branching logics and adaptive questioning, and assumes the respondent works in the pre-operative clinical area. The exported survey has been annotated to show all possible drop-down item selections.

Plain Language Summary/Consent

We are conducting this survey to understand the current practices across Australia and New Zealand in the exercise health professional (e.g. physiotherapist, exercise physiologist, allied health assistant, etc.) assessment and management of people undergoing lung cancer surgery in the pre-operative and post-operative phase (up to 12 months after surgery).

Please read the Plain Language Statement prior to commencing. If you have any questions or concerns regarding this survey, please contact Georgina Whish-Wilson on g.whishwilson@student.unimelb.edu.au or +613 8344 8126, or A/Prof Catherine Granger on catherine.granger@unimelb.edu.au or +613 8344 8126.

By selecting 'agree' below, you are acknowledging that you have read and understood the information above, and that you wish to participate.

- ○ Yes, I agree.
- ○ No, I do not agree and do not wish to continue with the survey.

Eligibility

The following questions are to assess your eligibility to complete this survey, and are being asked to ensure the data collected by this survey is representative of the current workforce. This information will not be used in the data analysis, shared or published in any way.

Are you an allied health professional specialising in exercise-based interventions? E.g. Physiotherapist, Exercise Physiologist, Allied Health Assistant

- ○ Yes
- ○ No

Do you **currently** work in a setting that manages people undergoing lung cancer surgery, in any clinical setting (e.g. pre-operative exercise/assessment, acute wards/ICU,

community/outpatient programs)?

- Yes
- No

What is your full name?

What is the name of your workplace?

If you work across multiple workplaces, please select **one** and only refer to practices **at this workplace** throughout the survey. If you choose to, you can complete the survey multiple times to account for your other workplace(s).

What is your professional email address for this workplace?

Demographics

Please select your gender:

- Male
- Female
- Non-binary
- Prefer not to say
- Prefer to self-describe

Where do you currently work?

Drop-down box:
Australian Capital Territory
New South Wales
Northern Territory
Queensland
South Australia
Tasmania
Victoria
Western Australia
New Zealand

Please state the postcode of your primary place of clinical practice

[ ]

For how many years you been working as an exercise health professional?

[▾]

Drop-down box: Numbered options ranging from from less than 1 year to more than 15 years

What is your exercise health discipline?

- ○ Physiotherapist
- ○ Exercise Physiologist
- ○ Allied Health Assistant (please specify specialisation if applicable) [ ]
- ○ [ ] Other (please specify)

In which country did you complete your workforce entry qualification?

- ○ Australia
- ○ New Zealand
- ○ [ ] Other (please specify)

What is the highest level of education that you have completed?

- ○ Workforce entry diploma/certificate
- ○ Workforce entry degree (e.g. Bachelor's, Graduate entry Masters/Doctorate, etc.)
- ○ Post-graduate coursework specialisation degree (e.g. Masters via coursework - please [ ] specify)
- ○ Post-graduate research masters degree (e.g. Masters of Philosophy)
- ○ Post-graduate research doctorate degree (e.g. Doctor of Philosophy)
- ○ [ ] Other (please specify)

How many years of experience do you have working with people with lung cancer in any setting?

[▾]

Drop-down box:
Numbered options ranging from from less than 1 year to more than 15 years

Please answer the following questions based on your current role(s) at your health service.

Please allocate 100 points to reflect the percentage of your work week allocated to clinical vs. non-clinical activities:

Clinical	0
Non-Clinical	0
Total	0

In which setting(s) do you currently work?

- ☐ Public Hospital
- ☐ Private Hospital
- ☐ Private Practice
- ☐ Pulmonary Rehabilitation
- ☐ ________ Other Public Community Setting (please specify)
- ☐ ________ Other (please specify)

At which time point do you <u>mainly</u> work with people undergoing lung cancer surgery? If you work across multiple settings, you will have the opportunity to select another time point at the end of the survey.

- ○ Pre-Operative (e.g. Pre-operative exercise/prehabilitation, pre-assessment clinic, etc.)
- ○ Post-Operative Acute (e.g. Surgical ward, ICU, etc.)
- ○ Community/Outpatient (e.g. Pulmonary rehabilitation, oncology rehabilitation, etc.)

How often do you receive referrals for patients with lung cancer (both operable and/or inoperable)?

- ○ Very often (once a week or more)
- ○ Often (once a fortnight)
- ○ Sometimes (once a month)
- ○ Rarely (once every 6 months)
- ○ Very rarely (once a year or less)

How much do you believe that each of the following have influenced the way you assess and treat patients with lung cancer?

	Not at all	A little	Somewhat	A lot	Very much
Published journal articles	○	○	○	○	○
Text books	○	○	○	○	○
Established practice at your workplace	○	○	○	○	○
Personal experience	○	○	○	○	○
Postgraduate education (workshops, seminars, etc.)	○	○	○	○	○
Professional development	○	○	○	○	○
Workforce entry degree	○	○	○	○	○
Other (please specify) ________	○	○	○	○	○

Pre-Operative - Assessment

Please answer the following questions based on **current practices at your workplace** relating to exercise health professional (e.g. physiotherapist, exercise physiologist, allied health assistant) involvement in the **pre-operative** period for people undergoing lung cancer surgery.

In your experience, how long is the average window between initial lung cancer diagnosis and date of surgery at your health service?

- Less than 1 week
- Between 1-2 weeks
- Between 2-4 weeks
- Between 4-8 weeks
- More than 8 weeks
- Unsure
- Already Answered

In which **pre-operative** setting(s) do you currently work? Select all that apply.

- [ ] Pre-operative exercise/Prehabilitation
- [ ] Pre-Admission Clinic (PAC)
- [ ] ________ Other (please specify)

At your health service, what proportion of patients awaiting lung cancer surgery are assessed by exercise health professionals in the **pre-operative** period?

- All patients
- Most patients
- Some patients
- A few patients
- No patients
- Unsure

What are the **patient-related factors** that usually trigger exercise health professional assessment of patients awaiting lung cancer surgery in the **pre-operative** period at your health service? Select all that apply.

- [ ] Presence of respiratory co-morbidity
- [ ] Advanced age
- [ ] Presence of frailty
- [ ] Presence of pre-operative mobility concerns
- [ ] Poor functional test outcome (e.g. CPET, stair climb etc)

- [ ] No patient-related factors are related to exercise health professional involvement at this timepoint
- [ ] ______ Other (please specify)

What are the **other** factors that influence exercise health professional assessment of patients awaiting lung cancer surgery in the **pre-operative** period at your health service? Select all that apply.

- [ ] Staff availability
- [ ] Referral processes
- [ ] No other factors are related to exercise health professional involvement at this timepoint
- [ ] ______ Other (please specify)

Which of the following parameters do **you** routinely assess in patients awaiting lung cancer surgery in the **pre-operative** setting? Select all that apply.

- [ ] Thoracic Spine Range of Motion (ROM)
- [ ] Shoulder Range of Motion (ROM)
- [ ] Muscle Strength/Power
- [ ] Exercise Capacity
- [ ] Mobility/Physical Function
- [ ] Frailty
- [ ] Falls Risk
- [ ] Patient-Reported Outcome Measures (e.g. breathlessness or quality of life questionnaires, etc.)
- [ ] Other Assessments (e.g. lung function, respiratory or peripheral muscle ultrasound, etc).
- [ ] None of the above

How do you assess **thoracic spine range of motion** of patients awaiting lung cancer surgery in the **pre-operative** setting? Select all that apply.

- [ ] Goniometry
- [ ] Visual Observation/"Eyeball" measurements
- [ ] ______ Functional tasks (please specify)

- [ ] ____ Other (please specify)

How do you assess **shoulder range of motion** of patients awaiting lung cancer surgery in the **pre-operative** setting? Select all that apply.

- [ ] Goniometry
- [ ] Visual Observation/"Eyeball" measurements
- [ ] ____ Functional tasks (please specify)
- [ ] ____ Other (please specify)

How do you assess **muscle strength/power** of patients awaiting lung cancer surgery in the **pre-operative** setting? Select all that apply.

- [ ] ____ Manual Muscle Testing (MMT) (please specify muscle groups)
- [ ] ____ Dynamometry (please specify muscle groups)
- [ ] ____ Functional tasks (please specify)
- [ ] ____ Sit to Stand Test (please specify e.g. 5x, 60sec, 30sec)
- [ ] ____ Other (please specify)

How do you assess **exercise capacity** of patients awaiting lung cancer surgery in the **pre-operative** setting? Select all that apply.

- [ ] 6-Minute Walk Test (6MWT)
- [ ] Stair Climbing Test
- [ ] Incremental Shuttle Walk Test (ISWT)
- [ ] Endurance Shuttle Walk Test (ESWT)
- [ ] Cardiopulmonary exercise test (CPET) (laboratory based test)
- [ ] ____ Other (please specify)

How do you assess **mobility/physical function** of patients awaiting lung cancer surgery in the **pre-operative** setting? Select all that apply.

- [ ] Gait pattern observation
- [ ] Mobility status (e.g. gait aid use, level of independence)
- [ ] Ten-Metre Walk Test (10MWT)
- [ ] Timed Up and Go Test (TUGT)
- [ ] Short Physical Performance Battery (SPPB)
- [ ] ____________ Other (please specify)

How do you assess **frailty** of patients awaiting lung cancer surgery in the **pre-operative** setting?

- [ ] Clinical Frailty Scale (CFS)
- [ ] Fried's Frailty Phenotype
- [ ] ____________ Other (please specify)

How do you assess **falls risk** of patients awaiting lung cancer surgery in the **pre-operative** setting? Select all that apply.

- [ ] Falls Risk Assessment Tool (FRAT)
- [ ] Falls Risk for Older People in the Community (FROP-Com)
- [ ] ____________ Balance assessments (please specify)
- [ ] ____________ Other (please specify)

Which **patient-reported outcome measures** do you administer to patients awaiting lung cancer surgery in the **pre-operative** setting? Select all that apply.

- [ ] Modified Medical Research Council Dyspnoea Scale (MMRC)
- [ ] Modified BORG Scale
- [ ] European Organisation for Research and Treatment of Cancer Quality of Life Questionnaire + Lung Module 13 (EORTC QLQ LC13)
- [ ] Functional Assessment of Cancer Therapy Lung Module (FACT-L)
- [ ] Chronic Respiratory Disease Questionnaire (CRDQ)
- [ ] St George's Respiratory Questionnaire (SGRQ)
- [ ] Medical Outcomes Study Short Form 36 (MOS SF 36)
- [ ] Brief Fatigue Inventory

☐ Cancer-Related Fatigue Distress Scale
☐ ______ Other (please specify)

Which **other assessments** do you administer to patients awaiting lung cancer surgery in the **pre-operative** setting? Select all that apply.

Please only select assessments that **you** specifically administer, rather than those completed by others that you may access/interpret.

☐ Lung Function Testing
☐ Lung Ultrasound
☐ Respiratory Muscle Ultrasound (e.g. diaphragm, intercostals)
☐ Peripheral Muscle Ultrasound (e.g. quadriceps, tibialis anterior)
☐ Please specify any other tests not captured above routinely assessed by exercise health ______ professionals:

Please allocate 100 points to reflect the percentage of involvement of each discipline in the exercise health professional assessment of patients awaiting lung cancer surgery in the **pre-operative** setting at your health service:

Physiotherapy	0
Exercise Physiology	0
Allied Health Assistant	0
Other (please specify) ______	0
Total	0

Pre-Operative - Management

Please answer the following questions based on current practices at your workplace relating to exercise health professional (e.g. physiotherapist, exercise physiologist, allied health assistant) involvement in the **pre-operative** period for people awaiting lung cancer surgery.

What proportion of the patients awaiting lung cancer surgery that you see in this period are provided with **pre-operative** education by an exercise health professional?

- ○ All patients
- ○ Most patients
- ○ Some patients
- ○ A few patients
- ○ No patients
- ○ Unsure

What proportion of patients awaiting lung cancer surgery are provided with **pre-operative** education by exercise health professionals at your health service relating to:

	None of them	A few of them	Some of them	Most of them	All of them
Breathing exercises	○	○	○	○	○
Cough/huff	○	○	○	○	○
The importance of early ambulation	○	○	○	○	○
The importance of upright positioning	○	○	○	○	○
Thoracic mobility exercises	○	○	○	○	○
Shoulder mobility exercises	○	○	○	○	○
Explanation of post-operative physiotherapy sessions	○	○	○	○	○
Support services available on discharge	○	○	○	○	○
Disease-specific exercise guidelines	○	○	○	○	○
Other (please specify) ______	○	○	○	○	○

Please allocate 100 points to reflect the percentage of involvement of each discipline in the delivery of **pre-operative** eduction to patients awaiting lung cancer surgery at your health service:

Physiotherapy	0
Exercise Physiology	0
Allied Health Assistant	0
Other (please specify)	0
Total	0

What proportion of the patients awaiting lung cancer surgery that you see in this period participate in a **pre-operative** exercise program?

- ○ All patients
- ○ Most patients
- ○ Some patients
- ○ A few patients
- ○ No patients
- ○ Unsure

Are there specific eligibility criteria for patients awaiting lung cancer surgery to participate in the **pre-operative** exercise program?

- ○ Yes
- ○ No

Please specify these eligibility criteria:

The following questions relate to the delivery of your **pre-operative** exercise program over the **last four weeks.**

What is the typical duration, frequency and intensity of the **pre-operative** exercise program?

Duration of program (in weeks) [ ]

Frequency (no. of sessions per week) [ ]

Length of sessions (in minutes) [ ]

Intensity [ ]

How is the **pre-operative** exercise program primarily delivered? Select all that apply.

- ☐ In-Person
- ☐ Remote - via Telephone
- ☐ [ ] Remote - via Telehealth/Video (please specify platform)
- ☐ [ ] Remote - via App (please specify platform)
- ☐ [ ] Other (please specify)

Where is the **pre-operative** exercise program primarily delivered?

- ○ Centre-Based (e.g. hospital, community gym, etc.)
- ○ Home-Based
- ○ [ ] Hybrid Model (please specify)
- ○ [ ] Other (please specify)

Do participants receive supervision during the **pre-operative** exercise program?

- ○ Yes, the program is supervised
- ○ No, the program is unsupervised
- ○ [ ] Hybrid Model (please specify)
- ○ [ ] Other (please specify)

Do participants attend the **pre-operative** exercise program individually or in a group?

- ○ One-on-One
- ○ Group
- ○ [ ] Hybrid Model (please specify)

○ [ ] Other (please specify)

Please outline any ways that COVID-19 restrictions have impacted the delivery of this service over the **last 4 weeks** compared to usual care/pre-COVID:

[ ]

What proportion of patients participating in the **pre-operative** exercise program are prescribed the following forms of exercise at your health service:

	None of them	A few of them	Some of them	Most of them	All of them
Walking	○	○	○	○	○
Cycling	○	○	○	○	○
Strengthening (upper limb)	○	○	○	○	○
Strengthening (lower limb)	○	○	○	○	○
Range of motion	○	○	○	○	○
Balance training	○	○	○	○	○
Hydrotherapy	○	○	○	○	○
Functional exercise	○	○	○	○	○
Breathing exercises	○	○	○	○	○
Inspiratory muscle training	○	○	○	○	○
Home exercise program	○	○	○	○	○
Other (please specify) [ ]	○	○	○	○	○

Please allocate 100 points to reflect the percentage of involvement of each discipline in the delivery of the **pre-operative** exercise program prior to lung cancer surgery:

Physiotherapy	0
Exercise Physiology	0
Allied Health Assistant	0
Other (please specify) [ ]	

Total 0

Are there any other non-exercise 'prehabilitation' components offered as part of your program? Select all that apply.

- ☐ Nutritional counselling
- ☐ Smoking cessation
- ☐ Psychological support
- ☐ No
- ☐ Unsure
- ☐ ______ Other (please specify)

Restart Survey #1

Do you work with people with people undergoing lung cancer surgery at an **additional time point** than previously mentioned and would like to complete another relevant section of the survey?

- ○ Post-Operative Acute (e.g. Surgical ward, ICU, etc.)
- ○ Community/Outpatient (e.g. Pulmonary rehabilitation, oncology rehabilitation, etc.)
- ○ No
