# Supplementary material for: Physiotherapy and Exercise Management of People Undergoing Surgery for Lung Cancer: A Survey of Current Practice across Australia and New Zealand
Source: J Clin Med. 2023 Mar 9;12(6):2146. doi: 10.3390/jcm12062146 (PMC10051547; doi:10.3390/jcm12062146)
Supplement: Supplementary file 1 [file jcm-12-02146-s001.zip › Supplementary Material 2.pdf]

Supplementary File S2: Reporting Checklists

Table S1. The Checklist for Reporting Results of Internet E-Surveys (CHERRIES)[1].

Item Category	Checklist Item	Page #	Description
**Design**	Describe survey design	2-3	An open, online survey distributed to allied health professionals specialising in exercise-based interventions, currently working in a setting that manages patients with lung cancer undergoing surgery, based in Australia and New Zealand.
**IRB (Institutional Review Board) approval and informed consent process**	IRB approval	13	The study had local university ethical approval (project ID 23112, 02/03/2022).
	Informed consent	2	Participants were displayed a detailed plain language summary on the opening page of the survey, containing information regarding the study aims, estimated length of the survey, data storage and privacy, and investigators. Informed consent was then collected via a tick-box.
	Data protection	3	Data was stored in a secure, password-protected, University-hosted server accessible only to the research team. Data was de-identified prior to analysis, and all re-identifiable data was stored separately to the main data file.
**Development and pre-testing**	Development and testing	2	An extensive three-staged testing process was adopted. Reviewers who were demographically similar to the target population provided feedback on the content, usability and technical functionality of the questionnaire prior to dissemination.
**Recruitment process and description of the sample having access to the questionnaire**	Open survey versus closed survey	2	This was an open survey. Eligibility was assessed within the opening pages of the survey.
	Contact mode	3	The physiotherapy and/or allied health managers of identified health services with thoracic surgery departments were emailed the survey link directly and asked to disseminate the survey to eligible clinicians.
	Advertising the survey	3-4	Aside from email dissemination, the survey was announced via multiple avenues including collaborative networks and via social media (Figure 1,

			Main Article). The social media announcement used is available in Supplementary File 3.
**Survey administration**	Web/E-mail	2	The survey was web-based, conducted via the online survey platform Qualtrics. Responses were automatically collated by the platform and exported for analysis.
	Context	3-4<br>& 2, Supplementary File 2	The online collaborative networks and social media groups (hosted via Facebook) used to distribute the survey were selected to extend our sample size and reach outside of the acute hospital setting (Figure 1, Main Article). These groups exist as avenues for exercise health clinicians with specific interests to share information, resources, and research projects. The groups chosen were relevant to the survey inclusion criteria (e.g., pulmonary rehabilitation, cancer, and cardiorespiratory interest groups). Given the target demographic for this survey were exercise health clinicians working in these clinical areas, we do not expect the use of these groups to have influenced the results.
	Mandatory/voluntary	2, Supplementary File 2	Participation in the survey was voluntary.
	Incentives	3	As an incentive to participate in the study, participants were eligible to enter a draw to win a new Apple iPad upon completing the survey. This was facilitated through a separate process that could not be linked to the study (i.e., participants were able to enter the draw via an entirely separate survey instrument and data were stored separately).
	Time/Date	3	The survey was open for five weeks between June-July 2022.
	Randomization of items or questionnaires	2, Supplementary File 2	The survey items were not randomised or alternated.
	Adaptive questioning	2	Adaptive questioning and branching logics were utilised throughout the survey, whereby only certain items or sections were displayed based on responses to other items this reduced the number and complexity of questions (e.g., only clinicians who reported assessing exercise capacity were then asked to identify the assessment tools used to assess exercise capacity).
	Number of Items	2	The total number of items varied widely depending on branching logic and adaptive questioning. The survey was divided into three main

			sections: 1) eligibility screening (6 items), 2) demographics (14 items), and 3) current clinical practice questions focused on elements of assessment, treatment, and education (32-34 items per timepoint [pre-operative, post-operative, and community/outpatient post discharge]).
	Number of screens (pages)	3, Supplementary File 2	The total number of screens/pages varied widely dependent on branching logic and adaptive questioning. The maximum number of pages displayed to a participant was 12.
	Completeness check	3, Supplementary File 2	All survey items were mandatory, and participants were unable to progress to the next page without selecting an answer for each item. Most items, except those linked to branching logics, included an 'unsure' option. Incomplete surveys were automatically 'submitted' upon the closure of the survey.
	Review step	3, Supplementary File 2	Participants were able to review and change their answers via a 'back' button throughout the entire survey up until completion. Participants were also able to leave the survey and re-enter at a later time to complete and submit the survey. There was no 'review' step.
**Response rates**	Unique site visitor	3, Supplementary File 2	Due to limitations of the survey tool used, it was not possible to determine the number of unique site visitors.
	View rate (Ratio of unique survey visitors/unique site visitors)	4 (Figure 1) & 3, Supplementary File 2	Due to limitations of the survey tool used, it was not possible to determine the number of unique site visitors or unique survey visitors. Therefore, it was not possible to calculate the view rate. The survey was emailed directly to 72 health services, and Figure 1 of the main article provides a summary of the number of members of each online group the survey was advertised within.
	Participation rate (Ratio of unique visitors who agreed to participate/unique first survey page visitors)	3, Supplementary File 2	Due to limitations of the survey tool used, it was not possible to determine the number of unique survey visitors. Therefore, it was not possible to calculate the participation rate.
	Completion rate (Ratio of users who finished the survey/users who agreed to participate)	5	The completion rate was 77% (102/132), i.e., 30 respondents provided informed consent but did not progress to participate in the survey. The completeness rate was 85% (87/102).

**Preventing multiple entries from the same individual**	Cookies used	3, Supplementary File 2	No.
	IP check	3 & 3, Supplementary File 2	No. As the survey was distributed to clinicians working in hospitals/health services, IP check was not appropriate given the likelihood of shared servers/computers.
	Log file analysis	3 & 3, Supplementary File 2	Participants were asked to nominate their name, workplace, and professional email address to ensure response accountability and prevent multiple entries from the same individual. These were then checked manually by the research team. No duplicate responses from the same individual were identified.
	Registration	4, Supplementary File 2	No.
**Analysis**	Handling of incomplete questionnaires	3	Incomplete responses (i.e., surveys that were terminated early) were included in data analysis.
	Questionnaires submitted with an atypical timestamp	4, Supplementary File 2	No atypical timestamps were identified. The minimum length of time recorded for a 100% completed survey was approximately 7 minutes.
	Statistical correction	4, Supplementary File 2	Not applicable.

Table S2. Checklist for Reporting of Survey Studies (CROSS)[2].

Section/topic	Item	Item description	Reported on page #:
**Title and abstract**			
Title and abstract	1a	State the word "survey" along with a commonly used term in title or abstract to introduce the study's design.	1
	1b	Provide an informative summary in the abstract, covering background, objectives, methods, findings/results, interpretation/discussion, and conclusions.	1
**Introduction**			
Background	2	Provide a background about the rationale of study, what has been previously done, and why this survey is needed.	1-2
Purpose/aim	3	Identify specific purposes, aims, goals, or objectives of the study.	2
**Methods**			
Study design	4	Specify the study design in the methods section with a commonly used term (e.g., cross-sectional or longitudinal).	2
	5a	Describe the questionnaire (e.g., number of sections, number of questions, number and names of instruments used).	2
Data collection methods	5b	Describe all questionnaire instruments that were used in the survey to measure particular concepts. Report target population, reported validity and reliability information, scoring/classification procedure, and reference links (if any).	The instrument and target population are described on pages 2-3. Validity, reliability, scoring/classification, and reference links were not applicable.

	5c	Provide information on pretesting of the questionnaire, if performed (in the article or in an online supplement). Report the method of pretesting, number of times questionnaire was pre-tested, number and demographics of participants used for pretesting, and the level of similarity of demographics between pre-testing participants and sample population.	2
	5d	Questionnaire if possible, should be fully provided (in the article, or as appendices or as an online supplement).	Supplementary File 1
Sample characteristics	6a	Describe the study population (i.e., background, locations, eligibility criteria for participant inclusion in survey, exclusion criteria).	3
	6b	Describe the sampling techniques used (e.g., single stage or multistage sampling, simple random sampling, stratified sampling, cluster sampling, convenience sampling). Specify the locations of sample participants whenever clustered sampling was applied.	3
	6c	Provide information on sample size, along with details of sample size calculation.	The sample size is described on page 4. No sample size calculations were undertaken.
	6d	Describe how representative the sample is of the study population (or target population if possible), particularly for population-based surveys.	3, 5-6 (Table 1)
Survey administration	7a	Provide information on modes of questionnaire administration, including the type and number of contacts, the location where the survey was conducted (e.g., outpatient room or by use of online tools, such as SurveyMonkey).	2-3
	7b	Provide information of survey's time frame, such as periods of recruitment, exposure, and follow-up days.	3
	7c	Provide information on the entry process:	3
		For web-based surveys, provide approaches to prevent "multiple participation" of participants.	

Study preparation	8	Describe any preparation process before conducting the survey (e.g., interviewers' training process, advertising the survey).	3-4 (Figure 1)
Ethical considerations	9a	Provide information on ethical approval for the survey if obtained, including informed consent, institutional review board [IRB] approval, Helsinki declaration, and good clinical practice [GCP] declaration (as appropriate).	2, 14
	9b	Provide information about survey anonymity and confidentiality and describe what mechanisms were used to protect unauthorized access.	3
Statistical analysis	10a	Describe statistical methods and analytical approach. Report the statistical software that was used for data analysis.	4
	10b	Report any modification of variables used in the analysis, along with reference (if available).	N/A
	10c	Report details about how missing data was handled. Include rate of missing items, missing data mechanism (i.e., missing completely at random [MCAR], missing at random [MAR] or missing not at random [MNAR]) and methods used to deal with missing data (e.g., multiple imputation).	3, 5
	10d	State how non-response error was addressed.	N/A
	10e	For longitudinal surveys, state how loss to follow-up was addressed.	N/A
	10f	Indicate whether any methods such as weighting of items or propensity scores have been used to adjust for non-representativeness of the sample.	N/A
	10g	Describe any sensitivity analysis conducted.	N/A
**Results**			
Respondent characteristics	11a	Report numbers of individuals at each stage of the study. Consider using a flow diagram, if possible.	4 (Figure 1)

	11b	Provide reasons for non-participation at each stage, if possible.	4 (Figure 1)
	11c	Report response rate, present the definition of response rate or the formula used to calculate response rate.	3, 5
	11d	Provide information to define how unique visitors are determined. Report number of unique visitors along with relevant proportions (e.g., view proportion, participation proportion, completion proportion).	Unique visitors, view proportion and participation proportion could not be calculated. Completion rate and completeness rate are defined and calculated on pages 3 and 5 respectively.
Descriptive results	12	Provide characteristics of study participants, as well as information on potential confounders and assessed outcomes.	5 (Table 1)
Main findings	13a	Give unadjusted estimates and, if applicable, confounder-adjusted estimates along with 95% confidence intervals and p-values.	5-11
	13b	For multivariable analysis, provide information on the model building process, model fit statistics, and model assumptions (as appropriate).	N/A
	13c	Provide details about any sensitivity analysis performed. If there are considerable amount of missing data, report sensitivity analyses comparing the results of complete cases with that of the imputed dataset (if possible).	N/A
**Discussion**			
Limitations	14	Discuss the limitations of the study, considering sources of potential biases and imprecisions, such as non-representativeness of sample, study design, important uncontrolled confounders.	13
Interpretations	15	Give a cautious overall interpretation of results, based on potential biases and imprecisions and suggest areas for future research.	11-13
Generalizability	16	Discuss the external validity of the results.	13

Other sections			
Role of funding source	17	State whether any funding organization has had any roles in the survey's design, implementation, and analysis.	14
Conflict of interest	18	Declare any potential conflict of interest.	14
Acknowledgements	19	Provide names of organizations/persons that are acknowledged along with their contribution to the research.	1, 14

References

1. Eysenbach, G., *Improving the quality of Web surveys: the Checklist for Reporting Results of Internet E-Surveys (CHERRIES).* Journal of medical Internet research, 2004. **6**(3): p. e34-e34.
2. Sharma, A., et al., *A Consensus-Based Checklist for Reporting of Survey Studies (CROSS).* Journal of General Internal Medicine, 2021. **36**(10): p. 3179-3187.
