# Supplementary material for: Physiotherapy and Exercise Management of People Undergoing Surgery for Lung Cancer: A Survey of Current Practice across Australia and New Zealand
Source: J Clin Med. 2023 Mar 9;12(6):2146. doi: 10.3390/jcm12062146 (PMC10051547; doi:10.3390/jcm12062146)
Supplement: Supplementary file 1 [file jcm-12-02146-s001.zip › Supplementary Material 3.pdf]

Supplementary File S3: Social Media Survey Announcement Example

The following announcement was published on social media:

"Go into the draw to win a new Apple iPad and help us to understand current practices around exercise management for people undergoing thoracic surgery for lung cancer in Australia or New Zealand!

Researchers from the University of Melbourne want to hear from you regarding the current practices in the assessment and management of these patients across the continuum (e.g., pre-operatively, post-operative acute, and outpatient/community programs including pulmonary and oncology rehabilitation).

Link to survey and plain language statement here: bit.ly/thoracicsurgsurvey

We would appreciate it if you could share this widely among your networks (not just physios) so that we can get an accurate snapshot of current clinical practice in this area.

Please email Ms Georgina Whish-Wilson (gwhishwilson@student.unimelb.edu) PhD student with any queries.

Thank you!"

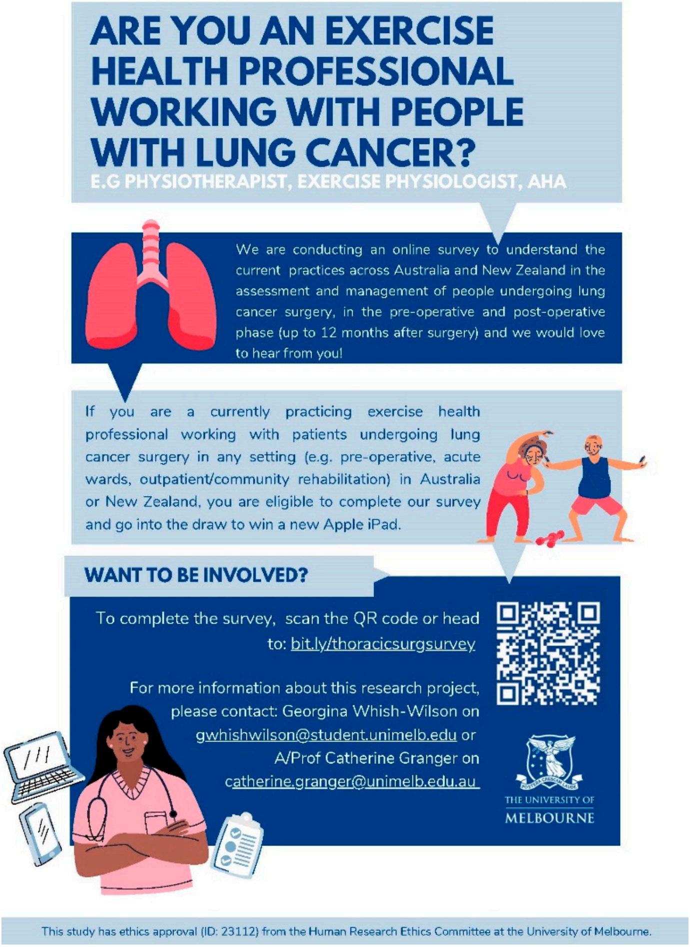

Figure S1. Flyer attached to social media post advertising the survey.
