# Supplementary material for: Physiotherapy and Exercise Management of People Undergoing Surgery for Lung Cancer: A Survey of Current Practice across Australia and New Zealand
Source: J Clin Med. 2023 Mar 9;12(6):2146. doi: 10.3390/jcm12062146 (PMC10051547; doi:10.3390/jcm12062146)
Supplement: Supplementary file 1 [file jcm-12-02146-s001.zip › Supplementary Material 4.pdf]

Supplementary File S4: Outcome Measures Utilised Across the Continuum

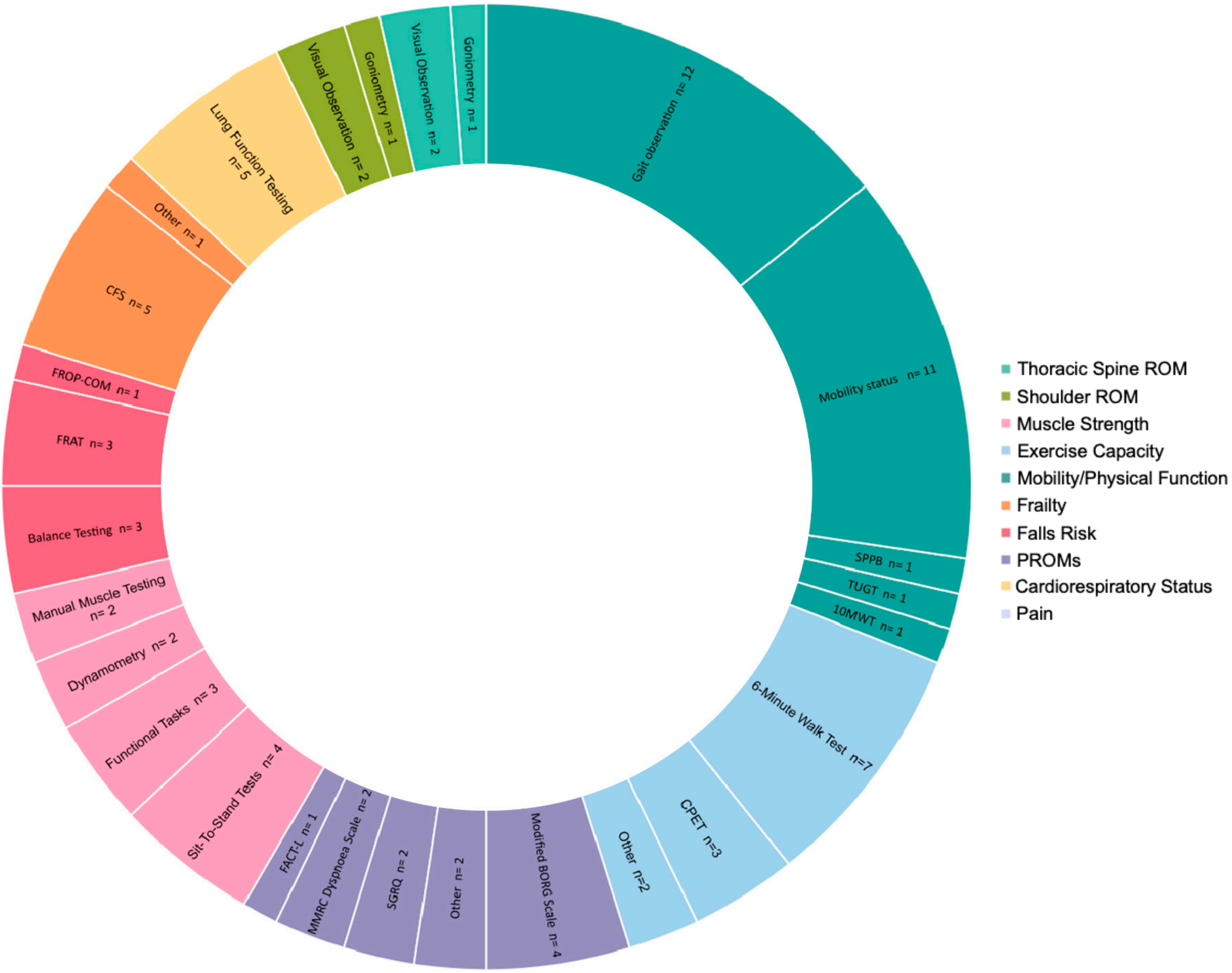

Figure S2. Number of respondents utilising outcome measures in the pre-operative timepoint (total n=17). Respondents could select more than one response for each parameter. Other frailty assessments reported: TUG (n=1); other exercise capacity assessments reported: subjective measures (n=2); other PROMs reported: EQ-5D (n=1), FACT-G (n=1).

Abbreviations:

ROM: Range of Motion

PROMs: Patient Reported Outcome Measures

CFS: Clinical Frailty Scale

FROP-COM: Falls Risk for Older People in the Community

FRAT: Falls Risk Assessment Tool

FACT-L: Functional Assessment of Cancer Therapy - Lung

MMRC: Modified Medical Research Council

SGRQ: St George's Respiratory Questionnaire

CPET: Cardiopulmonary Exercise Testing

10MWT: 10 Metre Walk Test

TUGT: Timed Up and Go Test

SPPB: Short Physical Performance Battery

FACT-G: Functional Assessment of Cancer Therapy - General

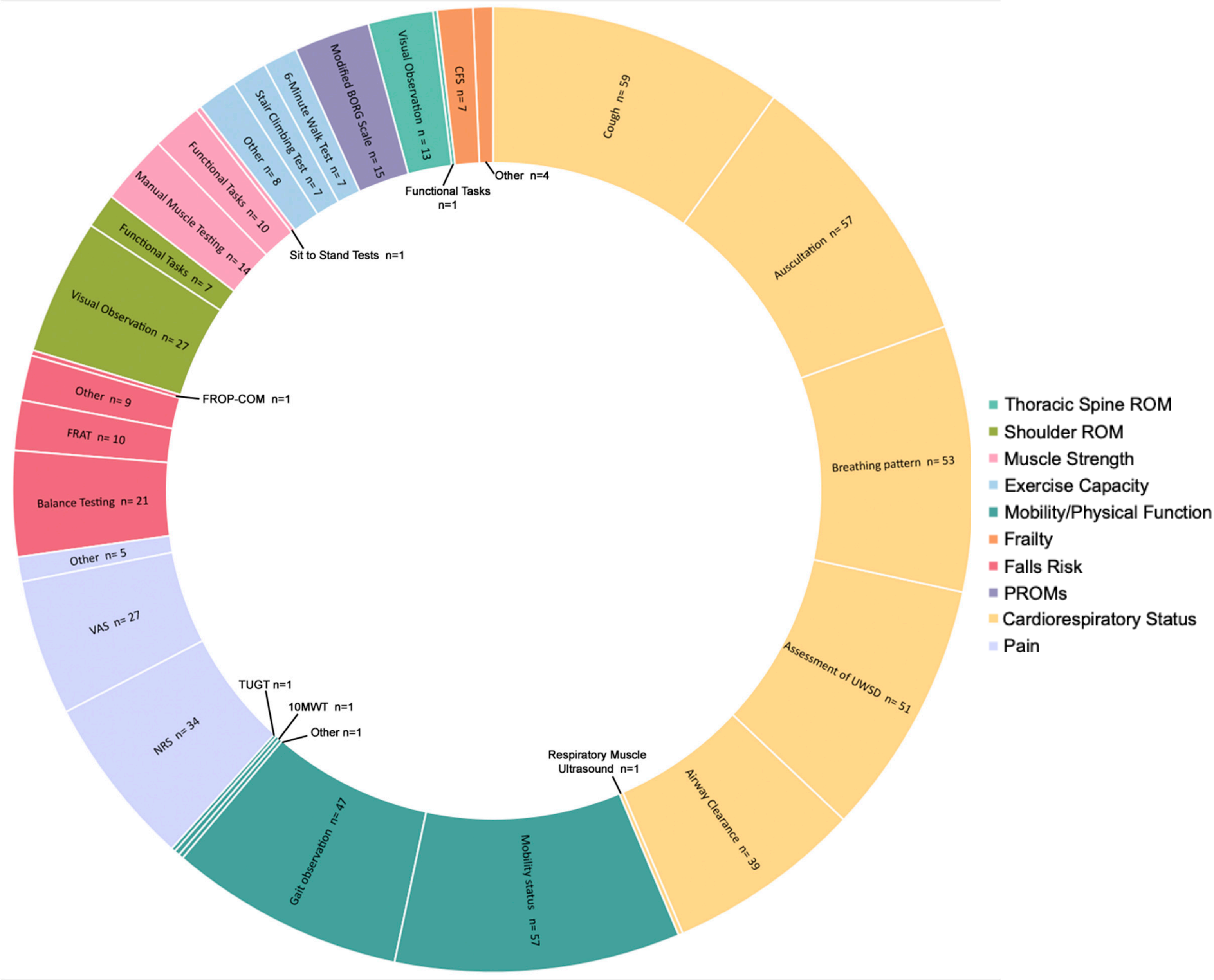

Figure S3. Number of respondents utilising outcome measures in the inpatient post-operative timepoint (total n=59). Respondents could select more than one response for each parameter. Other frailty assessments reported: informal/subjective measures (n=4); other exercise capacity assessments reported: informal/subjective measures (n=8); other falls risk assessments reported: FRAMP (n=2) informal/subjective measures (n=7); other pain assessments reported: subjective assessment (n=3), impact on functional activities (n=2); other mobility/physical function assessments reported: ICU mobility scale (n=1).

Abbreviations:

ROM: Range of Motion

PROMs: Patient Reported Outcome Measures

CFS: Clinical Frailty Scale

FROP-COM: Falls Risk for Older People in the Community

FRAT: Falls Risk Assessment Tool

VAS: Visual Analogue Scale

NRS: Numerical Rating Scale

TUGT: Timed Up and Go Test

10MWT: 10 Metre Walk Test

UWSD: Underwater Sealed Drain

FRAMP: Falls Risk Assessment and Management Plan

ICU: Intensive Care Unit

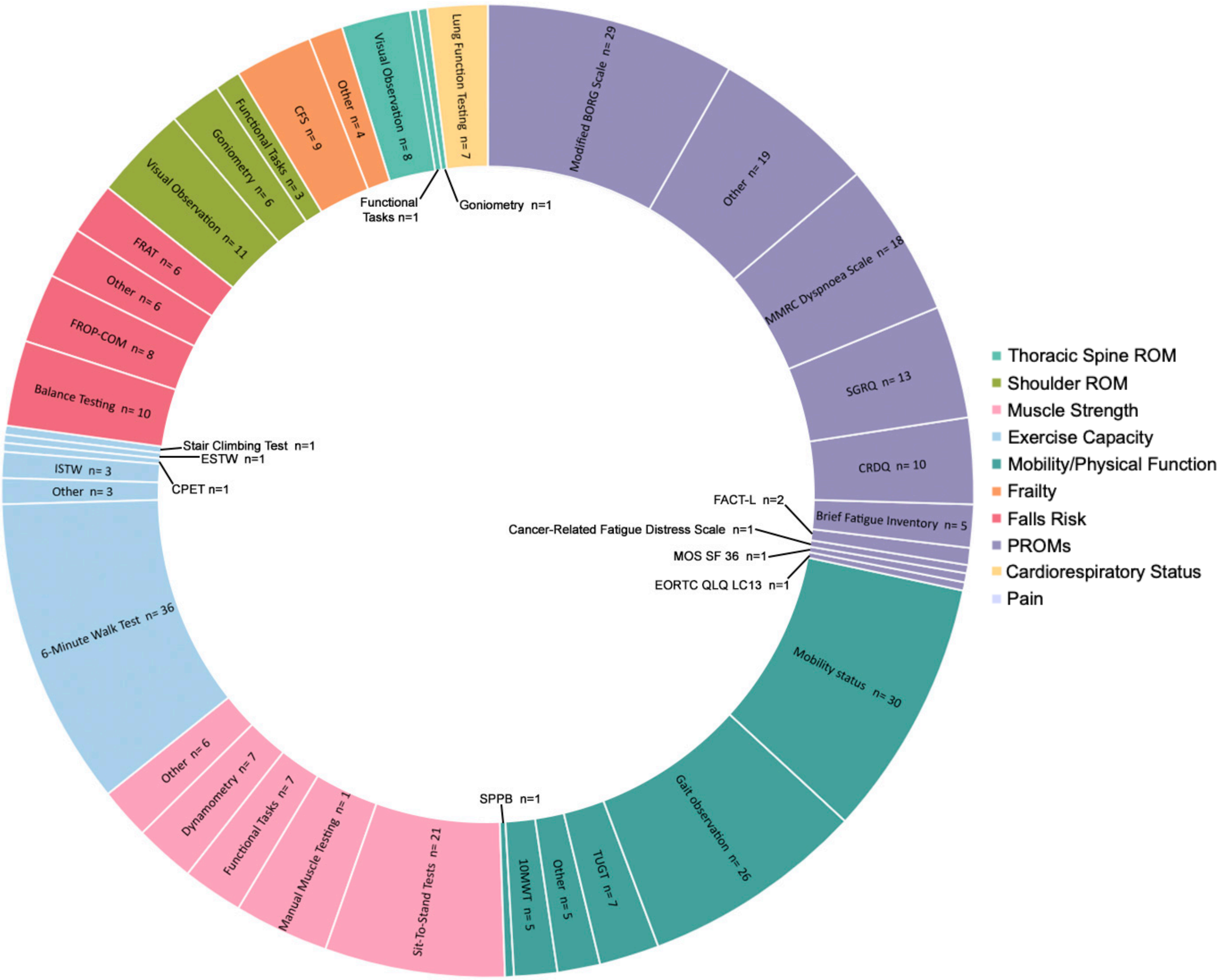

Figure S4. Number of respondents utilising outcome measures in the outpatient community timepoint (total n=38). Respondents could select more than one response for each parameter. Other frailty assessments reported: frail scale + nutrition scores + balance assessment (n=1), grip strength + sarcopenia + mobility + weight (n=1), liver frailty index (n=1), overall MDT assessment/screening tool (n=1); other falls risk assessments reported: falls history (n=5), informal/subjective measures (n=1); other exercise capacity assessments reported: 1-minute STS (n=2), 30-second STS (n=1); other muscle strength assessments reported: bicep curl 10RM (n=1), bicep curl 30-second (n=1), 10RM leg press (n=1), step test (n=1), other repetition max testing not specified (n=2); other mobility/physical function assessments reported: 4-square step test (n=1), 4MGS (n=1), 5x STS (n=1), 1-minute STS (n=1), subjective assessment (n=1); other PROMs reported: FACT-G (n=4), HADS (n=3), EQ-5D (n=2), CAT (n=2), PSFS (n=1), FACIT-F (n=1), PAM (n=1), DASS-21 (n=1), EORTC QLQ C30 (n=1), PHQ9 (n=1), Godin-Shepherd Leisure-Time Physical Activity Questionnaire (n=1), IPAQ (n=1).

Abbreviations:

CFS: Clinical Frailty Scale

FRAT: Falls Risk Assessment Tool

FROP-COM: Falls Risk for Older People in the Community

ESWT: Endurance Shuttle Walk Test

CPET: Cardiopulmonary Exercise Testing

ISWT: Incremental Shuttle Walk Test

SPPB: Short Physical Performance Battery

10MWT: 10 Metre Walk Test

TUGT: Timed Up and Go Test

EORTC QLQ LC13: European Organization for the Research and Treatment of Cancer Quality of Life Questionnaire Lung Cancer 13

MOS SF 36: Medical Outcomes Study 36-item Short Form Health Survey

FACT-L: Functional Assessment of Cancer Therapy – Lung

CRDQ: Chronic Respiratory Disease Questionnaire

SGRQ: St George's Respiratory Questionnaire

MMRC: Modified Medical Research Council

MDT: Multidisciplinary team

STS: Sit-to-stand

10RM: 10 Repetition Max

4MGS: 4-Metre Gait Speed Test

FACT-G: Functional Assessment of Cancer Therapy – General

HADS: Hospital Anxiety and Depression Scale (HADS)

CAT: Chronic Obstructive Pulmonary Disease (COPD) Assessment Tool

PSFS: Patient Specific Functional Scale

FACIT-F: Functional Assessment of Chronic Illness Therapy – Fatigue

PAM: Patient Activation Measure

DASS-21: Depression Anxiety Stress Scale

EORTC QLQ C30: European Organization for the Research and Treatment of Cancer Quality of Life Questionnaire

PHQ9: Patient Health Questionnaire

IPAQ: International Physical Activity Questionnaire
