# Supplementary material for: Physiotherapy and Exercise Management of People Undergoing Surgery for Lung Cancer: A Survey of Current Practice across Australia and New Zealand
Source: J Clin Med. 2023 Mar 9;12(6):2146. doi: 10.3390/jcm12062146 (PMC10051547; doi:10.3390/jcm12062146)
Supplement: Supplementary file 1 [file jcm-12-02146-s001.zip › Supplementary Material 5.pdf]

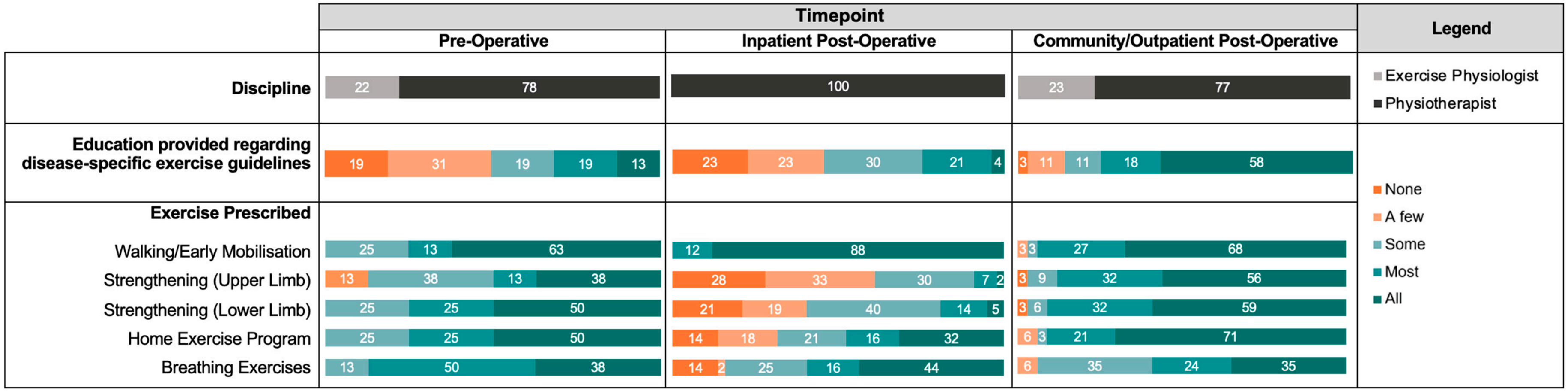

Figure S5. Comparison of exercise providers, education, and prescription across the continuum of care (% of respondents out of total).
